# Suplementação com Nitrito Atenua a Rigidez Vascular Independentemente do Exercício Físico

**DOI:** 10.36660/abc.20240836

**Published:** 2025-01-31

**Authors:** Alex Yuiti Ogura, Claudiane Maria Barbosa, Silvio Assis de Oliveira

**Affiliations:** 1 Universidade Federal de Mato Grosso do Sul Programa de Pós-Graduação em Ciências do Movimento Campo Grande MS Brasil Programa de Pós-Graduação em Ciências do Movimento (PPGCMov), Universidade Federal de Mato Grosso do Sul (UFMS), Campo Grande, MS – Brasil

**Keywords:** Remodelação Vascular, Nitritos, Exercício Físico, Rigidez Vascular

A prática de exercício físico é considerada uma das medidas não farmacológicas mais eficientes na redução da pressão arterial e no incremento do estado redox na parede de vasos sanguíneos.^1^ De fato, o exercício físico regular pode causar relaxamento do endotélio por meio do aumento dos níveis de óxido nítrico (NO), associado ou não com a redução na quantidade de espécies reativas de oxigênio (EROs). Produção e/ou excessivo acúmulo de EROs podem resultar em desequilíbrio redox, com diminuição da biodisponibilidade do NO e consequente disfunção endotelial.^1 , 2^ Nesse aspecto, menor liberação de NO configura um dos fatores críticos para desenvolvimento de hipertensão arterial sistêmica.^1 , 3^

De fato, o NO é uma molécula de sinalização gasosa diatômica que é solúvel em água e consegue passar livremente pela membrana celular. Esse peptídeo é fundamental na regulação de diversas ações fisiológicas no organismo, sendo de suma importância para processos de vasodilatação, resposta imune, neurotransmissão, apoptose e regulação genética.^4^ Quando liberado, o NO se difunde das células endoteliais para as células musculares lisas vasculares, promovendo relaxamento e vasodilatação.^5^ Por meio desse mecanismo, o NO pode reduzir a resistência periférica total e, consequentemente, diminuir a pressão arterial. No contexto endógeno, a produção de NO resulta da oxidação e clivagem do grupo guanidina da L-arginina, com liberação de NO e de L-citrulina. Em seguida, o NO livre é facilmente transformado em nitrito e nitrato, sendo estes produtos reciclados posteriormente em NO e outras formas de oxidação do nitrogênio, por meio da via nitrato/nitrito/NO.^6^

Na perspectiva exógena, o aporte de NO é resultante da alimentação. Em geral, 75% do nitrato advindo da alimentação é excretado na urina e o residual é absorvido pelas glândulas salivares que transformam nitrato em nitrito; na deglutição da saliva, o nitrito é protonado e se converte em ácido nitroso, o qual é também convertido em NO.^6^ Sendo assim, a ingestão de alimentos ricos nitrato e nitrito (beterraba, alface e espinafre), ou ainda, o consumo de suplemento específico têm potencial de aumentar a oferta de NO. Com a suplementação contínua, tem-se maior biodisponibilidade de NO, contribuindo para maior aporte de oxigênio e produção de ATP por meio da biogênese mitocondrial. Por conseguinte, há um impacto positivo quando se trata na melhora do desempenho na prática de exercício físico.^7^

Na presente edição dos Arquivos Brasileiros de Cardiologia, em Souza et al.,^8^ a suplementação oral com nitrito, de forma aguda, repercutiu em benefícios hemodinâmicos, como melhora da rigidez e pressão arterial. De fato, a rigidez arterial tem sido estudada em diferentes modelos experimentais,^9 , 10^ sendo considerada um importante preditor de risco cardiovascular. Neste mais novo estudo,^8^ a intervenção resultou em maiores níveis de nitrito no plasma, no músculo esquelético e no coração, de modo independente do exercício físico aeróbico. Portanto, o tratamento com nitrito pode ser uma alternativa para aumentar suas concentrações e, consequentemente, contribuir com maior biodisponibilidade do NO. Nesse contexto, a pressão arterial sistólica revelou-se reduzida em resposta ao nitrito e ao exercício físico de forma independente, sendo que a combinação das duas intervenções não resultou em benefícios adicionais. Além disso, a suplementação com nitrito demonstrou benefícios na remodelação cardíaca e no relaxamento vascular.

Uma possível fundamentação para esses achados é o aumento do fator de crescimento endotelial vascular, diminuição da concentração de EROs, aumento da produção e concentração de NO, redução na produção de Ang II e menor atividade simpática.^11 , 12^ Outros estudos mostraram que a suplementação oral de nitrito protege o endotélio vascular da atividade antioxidante, inibindo e ou reduzindo a atividade da NADPH oxidase e da xantina oxidoredutase em modelos animais.^13 , 14^ Portanto, a suplementação oral de nitrito, por se, promove múltiplos benefícios hemodinâmicos, como melhora da rigidez vascular, mostrando que, assim como a prática de exercício físico, pode ser uma alternativa para prevenção e tratamento da hipertensão arterial.
