# Supplementary material for: Suplementação com Nitrito Atenua a Rigidez Vascular Independentemente do Exercício Físico
Source: Arq Bras Cardiol. 2025 Jan 31;121(12):e20240836. [Article in Portuguese] doi: 10.36660/abc.20240836 (PMC12092037; doi:10.36660/abc.20240836)
Supplement: Translation

# Translation: Nitrite Supplementation Attenuates Vascular Stiffness Independently of Physical Exercise

**DOI:** 10.36660/abc.20240836i

**Authors:** Alex Yuiti Ogura, Claudiane Maria Barbosa, Silvio Assis de Oliveira

**Affiliations:** 1 Campo Grande MS Brazil Graduate Program in Movement Sciences (PPGCMov), Federal University of Mato Grosso do Sul (UFMS), Campo Grande, MS – Brazil

**Keywords:** Vascular Remodeling, Nitrites, Exercise, Vascular Stiffness

Exercise training is considered one of the most efficient non-pharmacological measures for reducing blood pressure and increasing the redox state in blood vessel walls.^1^Indeed, regular exercise training can cause endothelial relaxation by increasing nitric oxide (NO) levels, with or without a reduction in the amount of reactive oxygen species (ROS). Excessive production and/or accumulation of ROS can result in redox imbalance, with decreased NO bioavailability and consequent endothelial dysfunction.^1 , 2^In this regard, reduced NO release is one of the critical factors for the development of systemic arterial hypertension.^1 , 3^

Importantly, NO is a diatomic gaseous signaling molecule that is water-soluble and can freely pass through the cell membrane. This peptide is essential in the regulation of several physiological actions in the body, being of utmost importance for vasodilation, immune response, neurotransmission, apoptosis, and genetic regulation.^4^When released, NO diffuses from endothelial cells to vascular smooth muscle cells, promoting relaxation and vasodilation.^5^Through this mechanism, NO can reduce vascular peripheral resistance and, consequently, decrease blood pressure. In the endogenous context, NO production results from the oxidation and cleavage of the guanidine group of L-arginine, with the release of NO and L-citrulline. Then, free NO is easily transformed into nitrite and nitrate, the current products are later recycled into NO and other forms of nitrogen oxidation, through the nitrate/nitrite/NO pathway.^6^

From an exogenous perspective, the NO supplying could be result from food comsumption. Despite this, 75% of the nitrate from food is excreted in the urine, and the residual is absorbed by the salivary glands, which transform nitrate into nitrite; when swallowing saliva, nitrite is protonated and converted into nitrous acid, which is also converted into NO.^6^Therefore, the ingestion of foods rich in nitrate and nitrite (beets, lettuce, and spinach), or even the consumption of specific supplements, have the potential to increase the NO supplying. With continuous supplementation, there is greater bioavailability of NO, contributing to greater oxygen supply and ATP production through mitochondrial biogenesis. Consequently, there is a positive impact when it comes to improving performance in physical exercise.^7^

In the current issue of the *Arquivos Brasileiros de Cardiologia* , in Souza et al.,^8^acute oral supplementation with nitrite resulted in hemodynamic benefits, such as improved stiffness and blood pressure. Indeed, arterial stiffness has been studied in different experimental models^9 , 10^and is considered an important predictor of cardiovascular risk. In this most recent study,^8^the intervention resulted in higher levels of nitrite in plasma, skeletal muscle, and heart, independently of aerobic exercise. Therefore, treatment with nitrite may be an alternative to increase its concentrations and, consequently, contribute to greater bioavailability of NO. In this context, systolic blood pressure was shown to be reduced in response to nitrite and exercise training independently, and the combination of the two interventions did not result in additional benefits. Furthermore, nitrite supplementation demonstrated benefits in cardiac remodeling and vascular relaxation.

A possible explanation for these findings is the increase in vascular endothelial growth factor, decrease in ROS concentration, increase in NO production and concentration, reduction in Ang II production, and lower sympathetic activity.^11 , 12^Other studies have shown that oral nitrite supplementation protects the vascular endothelium from antioxidant activity, inhibiting and/or reducing the activity of NADPH oxidase and xanthine oxidoreductase in animal models.^13 , 14^Therefore, oral nitrite supplementation, by itself, promotes multiple hemodynamic benefits, such as improved vascular stiffness, showing that, like physical exercise, it may be an alternative for the prevention and treatment of arterial hypertension.
